# Comparability of activity monitors used in Asian and Western-country studies for assessing free-living sedentary behaviour

**DOI:** 10.1371/journal.pone.0186523

**Published:** 2017-10-18

**Authors:** Satoshi Kurita, Shohei Yano, Kaori Ishii, Ai Shibata, Hiroyuki Sasai, Yoshio Nakata, Noritoshi Fukushima, Shigeru Inoue, Shigeho Tanaka, Takemi Sugiyama, Neville Owen, Koichiro Oka

**Affiliations:** 1 Graduate School of Sport Sciences, Waseda University, Tokorozawa, Saitama, Japan; 2 Faculty of Sport Sciences, Waseda University, Tokorozawa, Saitama, Japan; 3 Faculty Health and Sport Sciences, University of Tsukuba, Tsukuba, Ibaraki, Japan; 4 Faculty of Medicine, University of Tsukuba, Tsukuba, Ibaraki, Japan; 5 Department of Preventive Medicine and Public Health, Tokyo Medical University, Shinjuku-ku, Tokyo, Japan; 6 Department of Nutritional Sciences, National Institute of Health and Nutrition, National Institutes of Biomedical Innovation, Health and Nutrition, Shinjuku-ku, Tokyo, Japan; 7 Institute for Health & Ageing, Australian Catholic University, Melbourne, Victoria, Australia; 8 Behavioural Epidemiology Laboratory, Baker IDI Heart and Diabetes Institute, Melbourne, Victria, Australia; 9 Swinburne University of Technology, Melbourne, Victoria, Australia; Centre National de la Recherche Scientifique, FRANCE

## Abstract

This study aims to compare the outputs of the waist-worn Active style Pro HJA-350IT (ASP; used in studies with Asian populations), the waist-worn ActiGragh^™^GT3X+ using the normal filter (GT3X+) and the thigh-worn activPAL3 (AP) in assessing adults’ sedentary behaviour (total sedentary time, number of breaks) under free-living conditions. Fifty healthy workers wore the three monitors simultaneously during their waking hours on two days, including a work day and a non-work day. Valid data were at least 10 hours of wearing time, and the differences between monitors on the sedentary outputs using the AP as criterion measurement were analyzed by ANOVA. The number of participants who had complete valid data for work day and non-work day was 47 and 44, respectively. Total sedentary time and breaks estimated by the AP were respectively 466.5 ± 146.8 min and 64.3 ± 24.9 times on the work day and 497.7 ± 138.3 min and 44.6 ± 15.4 times on the non-work day. In total sedentary time, the ASP estimated 29.7 min (95%CI = 7.9 to 51.5) significantly shorter than the AP on the work day but showed no significant difference against the AP on the non-work day. The GT3X+ estimated 80.1 min (54.6 to 105.6) and 52.3 (26.4 to 78.2) significantly longer than the AP on the work day and the non-work day, respectively. For the number of breaks from sedentary time, on both days, the ASP and the GT3X+ estimated significantly more than the AP: 14.1 to 15.8 times (6.3 to 22.5) for the ASP and 27.7 to 28.8 times (21.8 to 34.8) for the GT3X+. Compared to the AP as the criterion, the ASP can underestimate total sedentary time and the GT3X+ can overestimate it, and more so at the lower levels of sedentary time. For breaks from sedentary time, compared to the AP, both the GT3X+ the ASP can overestimate.

## Introduction

Since the harmfulness of sedentary behaviour was identified [1], reducing sedentary behaviour have been an important goal to enhance public health [2] because lifestyles are becoming increasingly less active [3]. With this goal in mind, there has been increasing research on the prevalence and determinants of sedentary behaviour and interventions to reduce sitting time [4–7]. When measuring sedentary behaviour, considering that this is ubiquitous in daily routines, device-based objective measures have certain advantages over self-report instruments [8, 9]. Such objective measures enable researchers to determine patterns characterized by total sedentary time and breaks in sedentary time.

So far, various devices have been used in studies, which makes it difficult to compare or synthesize findings from different countries or populations. More precise international comparability is important for global health promotion to estimate relative risk of sedentary behaviour among the countries or populations and identify country or population specific correlates of sedentary behaviour. These have been examined almost exclusively through the use of self-report instruments [10–12]. In this context, there is the need for a better understanding of the comparability between the outputs of the different activity monitors used in studies.

The activPAL3 (AP; PAL Technologies Ltd., Glasgow, UK) is an inclinometer, which is attached to wearer’s thigh, and is known to have high level of accuracy in characterizing posture—discriminating sitting/reclining, standing and stepping [13–15]. It is often used as a criterion measure of sedentary behaviour under free-living conditions [16]. The ActiGraph^™^ (ActiGraph LLC, Pensacola, Florida, USA) is a commonly-used brand of accelerometer for measuring physical activity and sedentary behaviour. One of the versions, GT3X+, can be worn on a variety of locations on the body (wrist, waist, arm, thigh or ankle), and a waist-worn GT3X+ has less accuracy than the AP in assessing sedentary behaviour [17, 18] but has been used in some cohort studies [19]. More recently, the Active style Pro HJA-350IT (ASP; Omron Health Care Co., Ltd., Kyoto, Japan), a waist-worn accelerometer released in 2008, has been used to assess sedentary behaviour in studies that have been conducted primarily with Asian populations [20–24].

The ASP is commonly available and affordable device, particularly in the context of the modest funding that can be available to researchers. The cost of the ASP is approximately $US150 per unit which is less expensive than the GT3X+ ($US250 per unit) and AP (approximately $US400 per unit). The ASP processes raw input signal using algorithms containing a specific equation for sedentary activities, which have been validated with the Douglas Bag method in a controlled laboratory setting [25, 26]. Although the output of waist-worn GT3X+ has been compared with that of AP [17, 18, 27, –29], which showed the GT3X+ estimates more sedentary time and breaks than the AP, there has been no research comparing ASP to these monitors in their ability to detect sedentary behaviour.

In order to compare or integrate findings from studies using different activity monitors, not only do the differences between monitors need to be better understood, but relative differences of each monitor against standard measurement should also be examined. Among the ASP, AP, and GT3X+, the AP is highly accurate in detecting free-living sedentary behaviour, and thus may be used as a criterion measure [8]. We compared outputs (total sedentary time, the number of breaks and sedentary bouts) of the ASP, waist-worn GT3X+ and AP in measuring free-living sedentary behavior, using the outputs of the AP as a reference standard.

## Methods

### Participants

To compare the outputs under various sedentary patterns, participants were workers engaged in different task types, and data were collected both on a work day and a non-work day. Previous studies have shown the sedentary patterns of work day and non-work day to be different [30, 31]. Fifty participants (27 men, age range 22 to 69 years) were recruited through opportunistic sampling at diverse workplaces in Tokyo from November, 2014 to June, 2015. They were 35 staff members from a hospital including physiotherapists and office workers, five manual laborers from a factory, one system engineer from a company and nine researchers and staffs from a university. Eligible participants were healthy, aged 20 years or older and full-time workers who work at least five days a week and eight hours a work day.

### Procedure

Participants were provided with the three monitors and a written description for wearing the monitors with a diary column to record monitor wear time. They were instructed how to wear the monitors simultaneously during waking time of two days (a work day and a non-work day) and to remove them during sleeping, water-based activities such as bathing or swimming and doing sports that can involve collision. They were also asked to record the date and time displayed in the liquid crystal screen of the ASP when wearing or removing monitors. The screen display is shown in a previously-published paper [25]. Participants were asked height, weight and occupational task type (sitting task/ standing task/ walking task/ physical labor task) by self-report, and body mass index (BMI) was calculated. After finishing data collection, participants returned the three monitors and their diary descriptions. The research protocol was approved by the Ethics Committee at the Waseda University. All participants were informed the purpose of this study and gave written consent.

### Instruments

The ASP is a tri-axial monitor (74 × 46 × 34 mm and 60 g including a battery that has a sampling rate of 32 Hz using 12 bit analog-to-digital (A/D) converter. Participants wore the ASP on the right or left side of the waist using an elastic belt that was changed by odd or even participant ID. The ASP was initialized to collect data in 10- and 60-sec epochs, and the data of 60-sec epochs were used in the analysis. ASP data with 10sec-epoch was not examined because no previous studies for adults have used that epoch duration and we needed to standardize the epoch for comparison purposes with the other monitors. The ASP estimates the intensity of activity by metabolic equivalents (METs) and defines sedentary behaviour as ≤1.5 METs. The CSV data files from the ASP were downloaded by Omron health management software BI-LINK for physical activity professional edition ver1.0 and then the files were processed by custom software (Custom-written Macro program for compiling data).

The GT3X+, released in September 2010, is small (46×33×15 mm) and lightweight (19g) tri-axial monitor that has a sampling rate from 30 to 100 Hz using 12 bit A/D converter. Participants attached the GT3X+ which was enclosed in a small pocket on elastic waist belt and placed in line with the axillary line of iliac crest which was the opposite side of the ASP. The GT3X+ was initialized to collect raw tri-axial acceleration signal at 30 Hz and processed into 60-sec epochs from vertical axis with and without low-frequency extension (GT3X+-Norm and GT3X+-LFE) using Actilife software version 6.10.4. The cutpoints to estimate sedentary behaviour were set 100 and 150 counts per min [17, 18]. This paper reports the results of the GT3X+-Norm-100 and those from different settings (GT3X+-Norm-150, LFE-100 and LFE-150) in the additional file because the GT3X+-Norm-100 has been used widely in previous research.

The activPAL3, released in December 2012, has small and thin shape (5×35×7 mm) and lightweight (15g) tri-axial monitor which has a sampling rate of 20 Hz using 8 bit A/D converter. The AP was taped to the midline of the anterior surface of the right thigh using a square of pad tape for medical care (Hakujuji Co., Ltd., Tokyo, Japan). The software (PAL Analysis version 7.2.32) processed raw data and then exported time-stamped “event” data file to calculate sedentary variables. Sedentary bouts were determined by any code of sitting/reclining (AP) similarly with previous studies.

### Data management

Reported time of wearing or removing monitors was used as rough indicator of start and stop time, and wear time was defined by the start and stop time of the ASP. The valid data was at least 600 min of wear time during 24 hours starting from midnight to midnight excluding non-wear time which was recorded in the column of the description by participants. In case the code the AP recorded was different from those of the ASP and GT3X+ on most of wear time, the data of the AP was judged as malfunctioning. In a previous study, three out of 26 APs malfunctioned, recording sitting during entire data collection (Steeves, 2015). The three sedentary outputs were as follows:

- Total sedentary time (min) ― this was the sum of the minutes when the monitors estimated sedentary behaviour.
- The number of breaks ― this was any interruptions in sedentary time which were counted when a transition occurred from a minute recorded as sedentary to an adjacent following minute recorded not sedentary: >1.5 METs in the ASP, ≥100 or ≥150 counts per min in the GT3X+, code of standing or stepping in the AP.
- The number of sedentary bouts ―a sedentary bout was defined as from starting to ending within sedentary time period. The number of sedentary bouts ≥2, ≥5, ≥10, ≥20, ≥30 and ≥60 min were calculated from each monitor.

### Statistical analysis

The variables on work day, non-work day and total (the mean of work day and non-work day) were separately analyzed. The differences of the outputs between monitors were examined using one-way repeated ANOVA with Tukey's post-hoc test. Overall magnitude correlation of sedentary time and breaks among monitors were examined by Pearson's correlation coefficient. In addition, Bland-Altman plots were created to evaluate the bias and limits of agreement and systematic error of total sedentary time and breaks against the AP as criterion. Proportional bias was measured by Pearson's correlation coefficient. The plots of the work day were considered occupational task types. All statistical analyses were performed using IBM SPSS Statistics 22 software (IBM Japan Inc., Tokyo, Japan). Significant levels were p < 0.05.

## Results

The number of participants who had valid data for the total, the work day and the non-work day was 43, 47 and 44, respectively. Some data were excluded due to lack of the data from the GT3X+or AP (n = 1 of work day, n = 1 of non-workday), lack of wear time (n = 2 of non-work day), configuration error of the GT3X+ or AP (n = 1 of work day, n = 1 of non-work day) and malfunctioning of the AP (n = 1 of work day, n = 2 of non-work day). Participants’ characteristics and descriptive statistics of the monitors’ outputs are summarized in Table 1 and S1 Table. Of the 47 sets of monitor data for work days, the number of sitting tasks, standing tasks, walking tasks and physical task at work was 19, 13, 11 and 4, respectively. Among participants who had the data of both work and non-work days, percent wear time of total sedentary time recorded by the AP of work day was lower than non-work day (53.1 ± 15.9% wear time for work days vs. 61.7 ± 15.7% wear time for non-work days, p<0.05).

**Table 1:** Participants' characteristics and descriptive statistics of monitors’ outputs.

	Total	Work day	Non-work day
**Participants**			
n (men)	43 (25)	47 (26)	44 (26)
Age	42.5 ± 12.2	41.6 ± 12.2	42.3 ± 12.2
Height (cm)	166.6 ± 8.5	166.0 ± 8.5	166.6 ± 8.4
Weight (kg) ^a^	61.2 ± 14.9	61.5 ± 11.7	62.6 ± 11.4
BMI (kg/m^2^) ^a^	21.9 ± 4.6	22.1 ± 3.2	22.3 ± 3.2
**Monitors’ outputs**			
Wear time (min/day)	846.8 ± 82.5	889.1 ± 103.3	803.9 ± 101.2
Total sedentary time (min/day)			
ASP	457.1 ± 97.9	436.8 ± 139.6	483.7 ± 140.4
GT3X+-Norm-100	546.4 ± 85.4	546.6 ± 125.2	550.0 ± 115.4
AP	482.7 ± 99.3	466.5 ± 146.8	497.7 ± 138.3
Total sedentary time (%wear time/day)			
ASP	53.9 ± 10.4	49.3 ± 15.2	60.0 ± 15.5
GT3X+-Norm-100	64.5 ± 8.0	61.6 ± 12.8	68.3 ± 10.7
AP	57.1 ± 10.8	52.5 ± 15.6	62.1 ± 15.8
Breaks (times/day)			
ASP	69.8 ± 14.1	78.3 ± 20.8	60.4 ± 19.2
GT3X+-Norm-100	83.0 ± 16.7	91.9 ± 24.2	73.3 ± 19.9
AP	55.2 ± 15.9	64.3 ± 24.9	44.6 ± 15.4
No. of sedentary bouts ≥2 min (times/day)			
ASP	44.7 ± 8.3	49.7 ± 13.3	39.8 ± 11.5
GT3X+-Norm-100	55.8 ± 11.1	61.1 ± 14.2	50.5 ± 14.2
AP	29.8 ± 7.1	34.6 ± 10.2	24.6 ± 6.6
No. of sedentary bouts ≥5 min (times/day)			
ASP	22.3 ± 4.8	23.3 ± 7.5	21.4 ± 6.2
GT3X+-Norm-100	28.8 ± 5.8	30.4 ± 7.7	27.5 ± 7.8
AP	19.3 ± 4.8	21.2 ± 6.5	17.2 ± 5.0
No. of sedentary bouts ≥10 min (times/day)			
ASP	11.5 ± 3.1	11.3 ± 4.8	11.9 ± 4.6
GT3X+-Norm-100	14.9 ± 3.3	15.0 ± 5.5	15.0 ± 4.3
AP	12.5 ± 3.4	13.1 ± 5.2	11.9 ± 3.5
No. of sedentary bouts ≥ 20 min (times/day)			
ASP	5.2 ± 2.0	4.4 ± 2.5	6.3 ± 3.3
GT3X+-Norm-100	5.9 ± 2.1	5.2 ± 3.1	6.8 ± 3.4
AP	6.2 ± 2.0	5.7 ± 3.4	6.9 ± 2.8
No. of sedentary bouts ≥ 30 min (times/day)			
ASP	2.8 ± 1.6	2.1 ± 1.9	3.7 ± 2.6
GT3X+-Norm-100	3.2 ± 1.5	2.6 ± 2.0	3.9 ± 2.6
AP	3.8 ± 1.7	3.3 ± 2.6	4.4 ± 2.3
No. of sedentary bouts ≥ 60 min (times/day)			
ASP	0.8 ± 0.9	0.4 ± 0.8	1.2 ± 1.5
GT3X+-Norm-100	0.8 ± 0.8	0.6 ± 0.9	1.0 ± 1.1
AP	1.3 ± 0.9	0.7 ± 0.9	1.8 ± 1.4

ASP, Active style Pro HJA350-IT; AP, activPAL3; GT3X+, ActiGraghTMGT3X+

Data are presented as mean ± SD

^a^ A participant did not answer.

**Fig 1:**
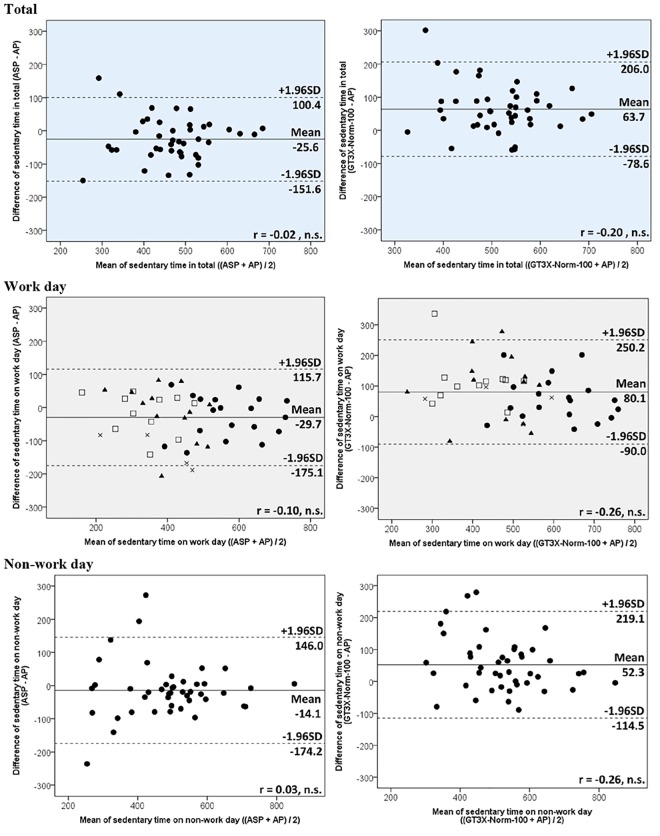
Bland-Altman plots of total sedentary time between monitors. The shape of the relationships on work days represent participants’ usual task at work; ●- sitting task, ▲- standing task, □- walking task, ×- physical task. Solid horizontal lines represent mean difference and dashed lines represent levels of agreement.

The differences for each of the outputs between the ASP and the other monitors are summarized in Table 2 and additional file 1 (S2 Table). For total sedentary time, overall, the ASP provided shorter time compared to the AP, while the GT3X+-Norm100 provided longer time. These differences were greater on the work day than on the non-work day. The ASP estimated 29.7 min (95%CI = 7.9 to 51.5) of total sedentary time significantly shorter than the AP on work day but showed no significant difference against the AP on non-work day. The GT3X+-Norm100 estimated 80.1 min (95%CI = 54.6 to 105.6) of total sedentary time and 52.3 min (95%CI = 26.4 to 78.2) significantly longer than the AP on work day and non-work day, respectively. The difference of total sedentary time between the ASP and GT3X+-Norm100 was more than one hour on both days. Overall magnitude correlations of total sedentary time among these monitors were significantly clearly shown (Table 3). Bland-Altman Plots of total sedentary time (Fig 1 and S1 Fig) indicated the ASP and GT3X+-Norm-100 had no systematic error against the AP in total. The participants’ usual task at work did not seem to affect the difference (middle of Fig 1 and S1 Fig).

**Table 2:** Mean differences in sedentary outputs between ASP, GT3X+-Norm-100, and AP by working status.

	Mean Difference (95% CI) ^a^		
	Total	Work day	Non-work day
Total sedentary time (min/day)			
ASP—AP	-25.6 (-45.4 to -5.8) *	-29.7 (-51.5 to -7.9) **	-14.1 (-38.9 to 10.8)
GT3X+-Norm-100—AP	63.7 (41.4 to 86.0) ***	80.1 (54.6 to 105.6) ***	52.3 (26.4 to 78.2) ***
GT3X+-Norm-100—ASP	89.3 (73 to 105.5) ***	109.8 (89.3 to 130.4) ***	66.4 (46.8 to 85.9) ***
Total sedentary time (%wear time/day)			
ASP—AP	-3.2 (-5.6 to -0.8) *	-3.2 (-5.7 to -0.8) **	-2.1 (-5.3 to 1.2)
GT3X+-Norm-100—AP	7.4 (4.8 to 10.1) ***	9.1 (6.2 to 12.0) ***	6.2 (3.0 to 9.3) ***
GT3X+-Norm-100—ASP	10.6 (8.6 to 12.6) ***	12.3 (10.1 to 14.6) ***	8.3 (5.8 to 10.7) ***
Breaks (times/day)			
ASP—AP	14.5 (8.7 to 20.3) ***	14.1 (6.3 to 21.9) ***	15.8 (9.2 to 22.5) ***
GT3X+-Norm-100—AP	27.8 (22.7 to 32.9) ***	27.7 (21.8 to 33.6) ***	28.8 (22.7 to 34.8) ***
GT3X+-Norm-100—ASP	13.3 (8.8 to 17.8) ***	13.6 (6.8 to 20.4) ***	12.9 (8.3 to 17.5) ***
No. of sedentary bouts ≥2 min (times/day)			
ASP—AP	14.9 (12.0 to 17.7) ***	15.2 (11.3 to 19.1) ***	15.1 (11.4 to 18.9) ***
GT3X+-Norm-100—AP	25.9 (23 to 28.9) ***	26.6 (23.5 to 29.7) ***	25.9 (22 to 29.9) ***
GT3X+-Norm-100—ASP	11.1 (8.2 to 14.0) ***	11.4 (7.3 to 15.5) ***	10.8 (7.6 to 13.9) ***
No. of sedentary bouts ≥5 min (times/day)			
ASP—AP	2.9 (1.6 to 4.3) ***	2.1 (0.4 to 3.8) *	4.2 (2.4 to 6.1) ***
GT3X+-Norm-100—AP	9.5 (8.0 to 11.0) ***	9.2 (7.6 to 10.8) ***	10.3 (8.1 to 12.6) ***
GT3X+-Norm-100—ASP	6.6 (5.3 to 7.9) ***	7.1 (5.6 to 8.6) ***	6.1 (4.7 to 7.6) ***
No. of sedentary bouts ≥10 min (times/day)			
ASP—AP	-1.0 (-1.9 to -0.2) *	-1.8 (-2.8 to -0.8) ***	0.0 (-1.2 to 1.3)
GT3X+-Norm-100—AP	2.4 (1.5 to 3.3) ***	1.9 (0.8 to 3.0) ***	3.2 (2.0 to 4.3) ***
GT3X+-Norm-100—ASP	3.5 (2.7 to 4.3) ***	3.7 (2.8 to 4.7) ***	3.1 (2 to 4.3) ***
No. of sedentary bouts ≥20 min (times/day)			
ASP—AP	-1.0 (-1.5 to -0.5) ***	-1.3 (-2.0 to -0.6) ***	-0.5 (-1.2 to 0.1)
GT3X+-Norm-100—AP	-0.3 (-0.8 to 0.2)	-0.5 (-1.1 to 0.1)	-0.1 (-0.7 to 0.6)
GT3X+-Norm-100—ASP	0.7 (0.1 to 1.2) *	0.8 (0.2 to 1.4) *	0.5 (-0.3 to 1.3)
No. of sedentary bouts ≥30 min (times/day)			
ASP—AP	-0.9 (-1.4 to -0.5) ***	-1.2 (-1.8 to -0.6) ***	-0.7 (-1.1 to -0.2) **
GT3X+-Norm-100—AP	-0.6 (-1 to -0.1) *	-0.7 (-1.2 to -0.2) **	-0.5 (-1.0 to 0.1)
GT3X+-Norm-100—ASP	0.3 (-0.1 to 0.8)	0.5 (0.1 to 0.9) *	0.2 (-0.4 to 0.7)
No. of sedentary bouts ≥60 min (times/day)			
ASP—AP	-0.5 (-0.7 to -0.2) ***	-0.3 (-0.5 to -0.1) **	-0.7 (-1.1 to -0.3) **
GT3X+-Norm-100—AP	-0.5 (-0.7 to -0.2) ***	-0.1 (-0.4 to 0.1)	-0.8 (-1.1 to -0.4) ***
GT3X+-Norm-100—ASP	0.0 (-0.2 to 0.3)	0.1 (-0.1 to 0.4)	-0.1 (-0.5 to 0.3)

ASP, Active style Pro HJA350-IT; AP, activPAL3; GT3X+, ActiGragh^™^GT3X+

*p < 0.05,

**p < 0.01,

***p < 0.001

**Table 3:** Pearson's correlation coefficients to examine overall magnitude correlation of the sedentary time and breaks among monitors.

	Total	Work day	Non-work day
Total sedentary time			
ASP—AP	0.79**	0.87**	0.83**
GT3X+-Norm-100—AP	0.70**	0.81**	0.79**
GT3X+-Norm-100—ASP	0.84**	0.87**	0.89**
Breaks			
ASP—AP	0.21	0.34*	0.38*
GT3X+-Norm-100—AP	0.48**	0.67**	0.58**
GT3X+-Norm-100—ASP	0.56**	0.48**	0.70**

ASP, Active style Pro HJA350-IT; AP, activPAL3; GT3X+, ActiGragh^™^GT3X+

*p < 0.05,

**p < 0.01

**Fig 2:**
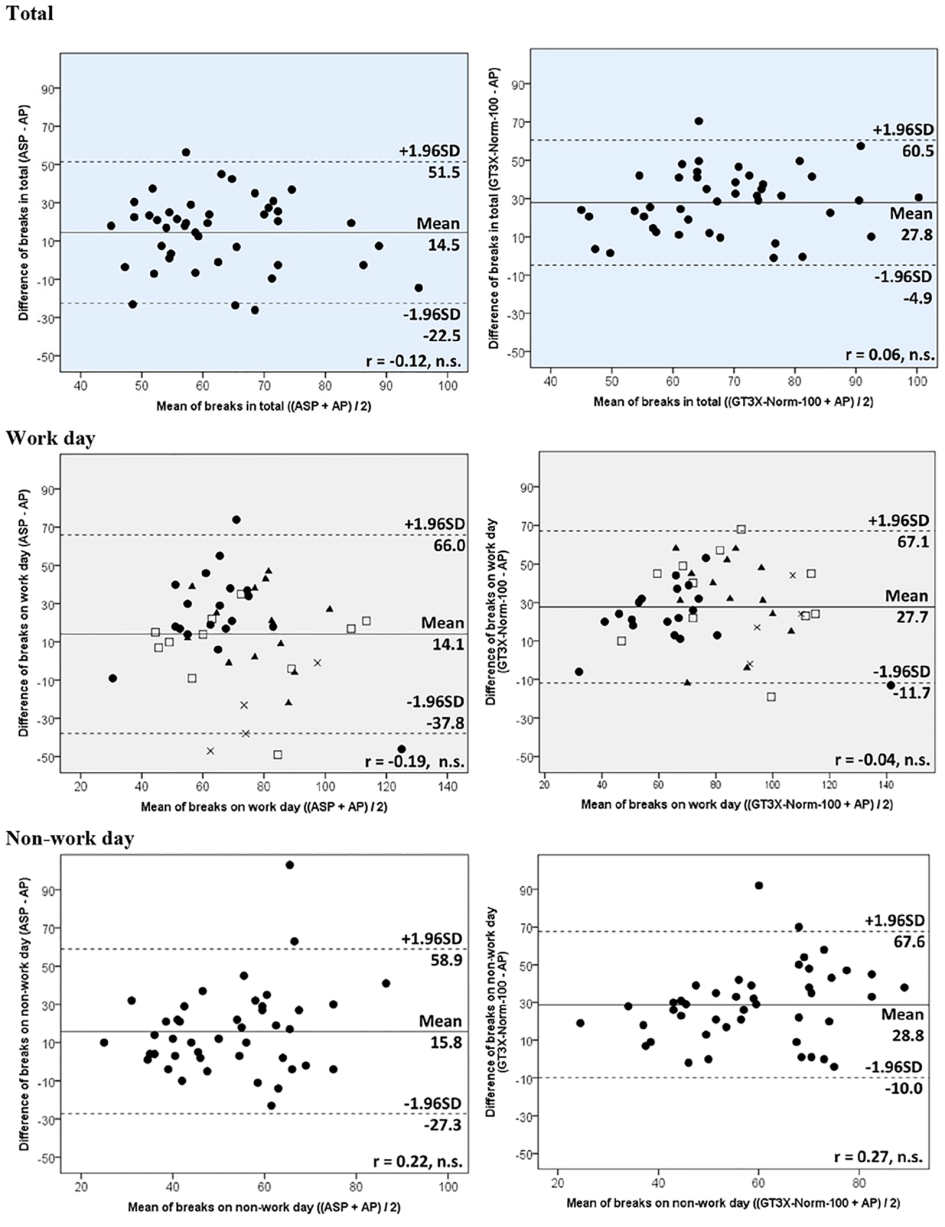
Bland-Altman plots of breaks between monitors. The shape of the relationships on work days represent participants’ usual task at work; ●- sitting task, ▲- standing task, □- walking task, ×- physical task. Solid horizontal lines represent mean difference and dashed lines represent levels of agreement.

For breaks from sedentary time, overall, the GT3X+-Norm100 followed by the ASP estimated more than the AP. In total, the ASP estimated 14.5 times (95%CI = 8.7 to 20.3) of breaks significantly more than the AP but 13.3 times (95%CI = 8.8 to 17.8) of breaks significantly less than the GT3X+-Norm-100. Overall magnitude of the correlations of breaks among between these monitors were less strong shown than total sedentary time, especially between the ASP and AP (Table 3). As with the total sedentary time, Bland-Altman plots of breaks (Fig 2 and S2 Fig) showed that the ASP and GT3X+ had no systematic error against the AP. The plots on the work day (middle of Fig 2 and S2 Fig) suggest that the GT3X+-Norm-100 estimated breaks of those whose work primarily involves walking tasks and physical labor tasks more than the ASP.

In the number of sedentary bouts, the GT3X+-Norm-100 followed by the ASP estimated a greater number of sedentary bouts ≥2 min and ≥5 min than did the AP. The ASP estimated significantly less bouts ≥10 min and ≥20 min than the AP, while the GT3X+-Norm-100 estimated significantly more bouts ≥10 min and equivalent bouts ≥20 min against the AP. The ASP and GT3X+-Norm-100 estimated significantly less bouts ≥30 and ≥60 min than the AP, but there were no significant differences between the ASP and GT3X+-Norm-100 in the bouts ≥60 min.

## Discussion

Comparing the outputs of the ASP, GT3X+ and AP in assessing sedentary behaviour under free-living condition, some key differences were observed between the outputs of the monitors that have been used in studies with Asian and Western samples. Participants were recruited from various occupations, so that participants except for those who have mainly sitting task were more sedentary on the non-work day than on the work day, and the present study was able to compare the outputs under measuring different sedentary patterns.

The main findings were that the ASP underestimated total sedentary time compared to the AP (Δ = -25.6 min/day, in total) and GT3X+-Norm-100 (Δ = -89.3 min/day, in total) with clear magnitude correlations. For sedentary breaks, the ASP output was greater than the AP (Δ = 14.5 times/day, in total) but less than the GT3X+ (Δ = -13.3 times/day, in total) with heterogeneous difference, especially between the ASP and AP. The differences and limits of agreement of total sedentary time and breaks of the ASP against the AP for non-work days were smaller than for work days, which indicates the estimation of the ASP for sedentary behaviour is more accurate when the sedentary level is relatively high, although the Bland-Altman plots showed there was no proportional bias. This tendency was similar with the GT3X+ and was consistent with the findings of a previous study [18]. The ASP tended to underestimate total sedentary time against the AP, probably because of the difference of epoch length (one minute for the ASP vs. 15 sec for the AP). It therefore seems that the AP may record shorter sedentary bouts lasting less than one minute. However, the difference of total sedentary time per day between ASP and AP was relatively small.

For the number of breaks, the ASP and GT3X+-Norm-100 overestimated against the AP. These tendencies were similar on both days. Previous studies have also reported the GT3X+-LFE-100 recorded breaks more than the AP [18, 28]. Lyden and colleagues [18] compared the number of breaks of the AP and GT3X (GT3X-Norm100, Norm150, LFE100 and LFE150) and showed there were differences (baseline condition: 0.3% vs. 77.8% to 110.7% bias for the AP and GT3X, respectively; and treatment condition: 10.9% vs. 98.1% to 133.3% bias for the AP and GT3X, respectively). Barreira et al. [28] also compared the number of breaks of the two monitors under free-living condition and reported the GT3X+ overestimated against the AP (39.0 ± 3.1 vs. 74.0 ± 4.1 for the AP and GT3X+, respectively). The heterogeneous and relatively large differences may be caused by the discrepancy of postural classification devices and energy-expenditure classification devices. The AP estimates the breaks from the angle of the thigh, while the ASP and the GT3X+ estimates from the acceleration of the waist motion. There were notable differences in the number of sedentary bouts ≥2, ≥5, ≥30 and ≥60 min between monitors which indicated the ASP and GT3X+-Norm-100 overestimated short sedentary bouts and underestimated prolonged sedentary bouts against the AP. This suggests the ASP and GT3X+ might estimate breaks during standing or sitting posture. The AP might classify sitting/lying even when activity while sitting or lying (such as fidgeting or changing posture) was more than 1.5 METs or 100 counts per min. To detect sedentary breaks accurately by waist-worn monitor is a future issue of this field. Advanced technology such as machine learning [32] has the potential in the future to resolve this problem. Interpretation with caution is essential in the results of previous epidemiologic studies on the association of breaks from sedentary time recorded by the waist-worn accelerometers with indicators of cardiometabolic risk [33–36].

The ASP may be able to classify sedentary behaviour and standing activity more accurately than does the GT3X+, because the GT3X+ overestimated breaks of most of those who were not primarily engaged in sitting tasks at work, compared to the ASP. Although no studies have examined how the ASP may misclassify static standing position as less or than 1.5 METs, Kerr et al. [37] reported that the GT3X+-Norm-100 recorded sedentary time during 72% of static standing position. Because the AP cannot accurately estimate the intensity of low intensity activities (sedentary to light) and the present findings showed heterogeneous difference between the ASP and AP, further research is needed to verify how accurately the ASP classifies standing and sitting time, by comparing these monitors under direct observation. The difference of the outputs between the ASP and GT3X+ may be explained mainly by the number of axis for sensing acceleration. The ASP estimate the intensity of the motion from triaxial information but the GT3X+ from uniaxial information, which is the same way with many previous studies. Using only the vertical axis may not be sensitive enough to measure low intensity physical activity even though the GT3X+ has low frequency extension setting. The GT3X+ may classify some light intensity activities as sedentary.

Overestimation of the GT3X+ against the AP in total sedentary time was also seen in a previous study. Two previous studies have compared the outputs of the AP and GT3X with low frequency extension. Lyden et al. [18] evaluated the validity of the AP and GT3X (LFE-100, LFE-150, Norm-100 and Norm-150) in measuring sedentary behaviour by direct observation with intervening participants to reduce sedentary behaviour. The GT3X overestimated total sedentary time against the AP in pre- and post- intervention (baseline condition: 1.6 vs. 5.6 to 17.8% for the AP and GT3X, respectively; and treatment condition: -0.1% vs. 35.9 to 50.7% bias for the AP and GT3X, respectively).

The main strength of our study is that it is the first to compare the outputs of the ASP (used primarily in studies with mainly Asian-country participant samples) and other monitors that have been used primarily with Western-country participant samples. Another strength is that this study compared the outputs of each monitor by measuring various activity patterns. We recruited men and women who engaged in various occupations and asked them to wear monitors on work day and non-work day. A limitation is the subjects were only healthy adults and small sample size, therefore the differences between monitors are unknown when they are used with other groups such as youth or older adults.

## Conclusions

In conclusion, the present findings demonstrate that the ASP, a device used mainly in Asian populations, can underestimate total sedentary time compared to the AP, while the GT3X+ can overestimate it against the AP. This tendency is more obvious when the sedentary time of participants is lower. For breaks from sedentary time, the GT3X+ followed by the ASP overestimated against the AP. Judging from the differences of the number of sedentary bouts against the AP, the ASP may misclassify sedentary breaks during sitting or standing posture, but the degree is lower than the GT3X+. These differences should be considered in sedentary behaviour research, especially in comparing Asian and Western study findings. Further research is needed to further clarify the differences between these monitors using direct observation as a criterion.

## Supporting information

S1 Fig Bland-Altman plots of total sedentary time between monitors. The shape of markers of work day represent participants’ usual task at work; ●- sitting task, ▲- standing task, □- walking task, ×- physical task. Solid horizontal lines represent mean difference and dashed lines represent levels of agreement.
(TIF)

S2 Fig Bland-Altman plots of breaks between monitors. The shape of markers of work day represent participants’ usual task at work; ●- sitting task, ▲- standing task, □- walking task, ×- physical task. Solid horizontal lines represent mean difference and dashed lines represent levels of agreement.
(TIF)

S1 Table Descriptive statistics of outputs of GT3X+-Norm-150, GT3X+-LFE-100 and GT3X+-LFE-150. (DOCX)

S2 Table Mean differences in sedentary outputs between monitors. Values [monitors (A) minus monitors (B)] are mean differences (95% CI). *p < 0.05, **p < 0.01, ***p < 0.001.
(DOCX)
