# Supplementary figures and images for: Comparability of activity monitors used in Asian and Western-country studies for assessing free-living sedentary behaviour
Source: PLoS One. 2017 Oct 18;12(10):e0186523. doi: 10.1371/journal.pone.0186523 (PMC5646850; doi:10.1371/journal.pone.0186523)

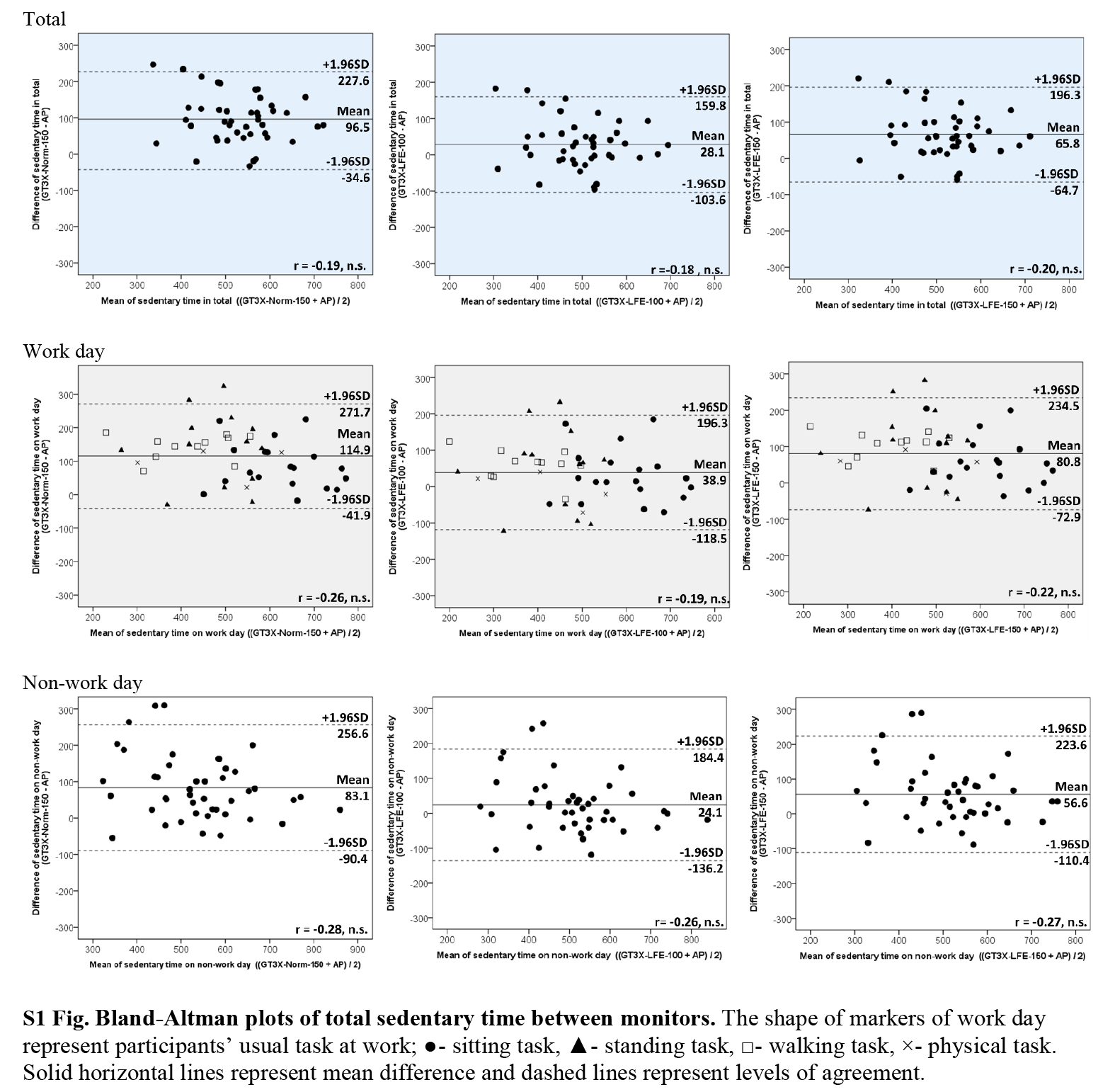

Supplement: S1 Fig — The shape of markers of work day represent participants’ usual task at work; ●- sitting task, ▲- standing task, □- walking task, ×- physical task. Solid horizontal lines represent mean difference and dashed lines represent levels of agreement. (TIF) [file pone.0186523.s001.tif]

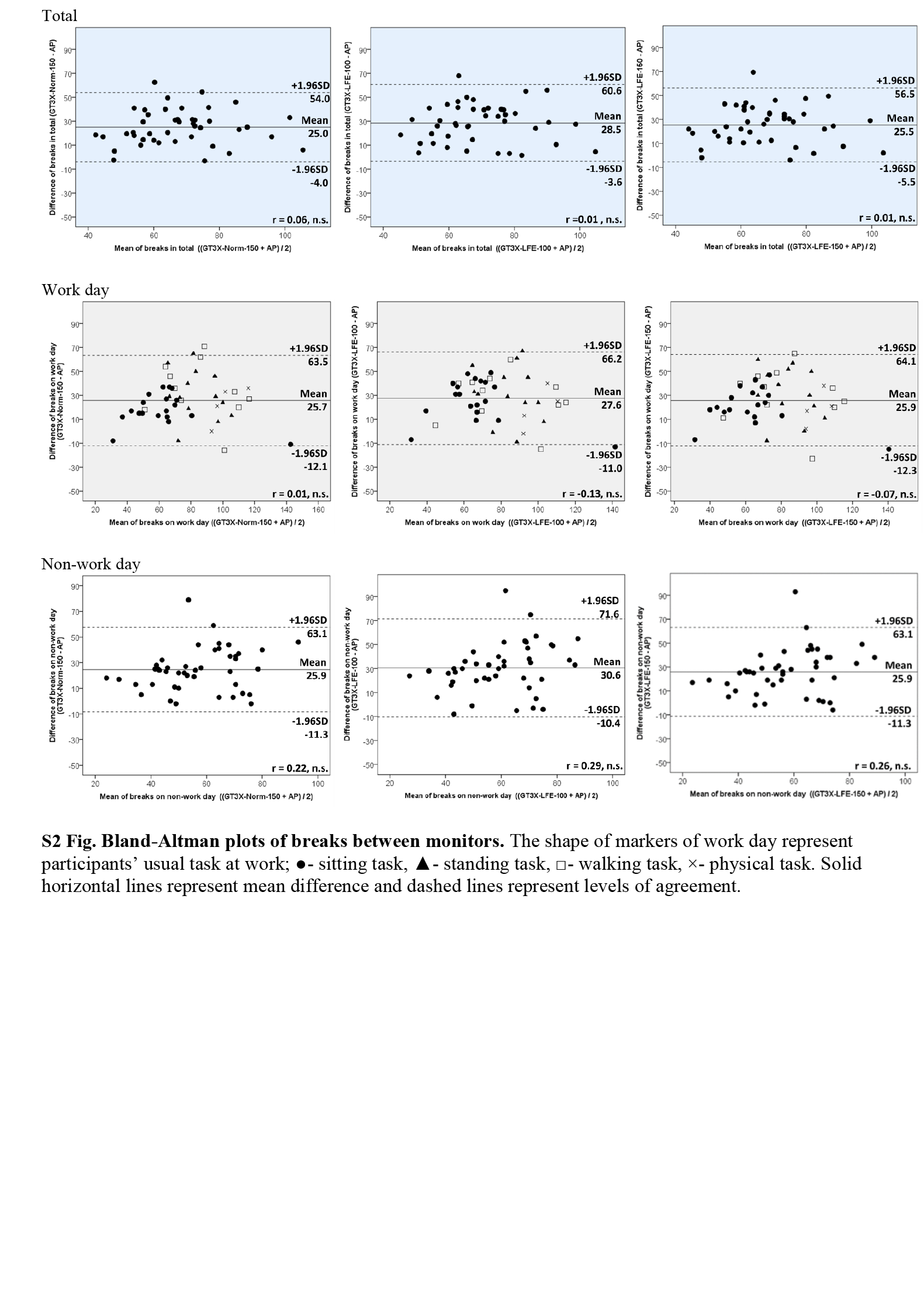

Supplement: S2 Fig — The shape of markers of work day represent participants’ usual task at work; ●- sitting task, ▲- standing task, □- walking task, ×- physical task. Solid horizontal lines represent mean difference and dashed lines represent levels of agreement. (TIF) [file pone.0186523.s002.tif]
