# Supplementary material for: Comparability of activity monitors used in Asian and Western-country studies for assessing free-living sedentary behaviour
Source: PLoS One. 2017 Oct 18;12(10):e0186523. doi: 10.1371/journal.pone.0186523 (PMC5646850; doi:10.1371/journal.pone.0186523)
Supplement: S1 Table — (DOCX) [file pone.0186523.s003.docx]

| **S1 Table. Descriptive statistics of outputs of GT3X+-Norm-150, GT3X+-LFE-100 and GT3X+-LFE-150** | | | | | |
| --- | --- | --- | --- | --- | --- |
|  | Total | | Work day | | Non-work day |
| **Monitors’ outputs** |  | |  | |  |
| Total sedentary time (min/day) | |  | |  |  |
| GT3X+-Norm-150 | 579.2 ± 87.4 | | 581.4 ± 126.8 | | 580.9 ± 115.2 |
| GT3X+-LFE-100 | 510.7 ± 88.1 | | 505.5 ± 132.3 | | 521.8 ± 117.9 |
| GT3X+-LFE-150 | 548.5 ± 86.9 | | 547.3 ± 130.1 | | 554.4 ± 116.4 |
| Total sedentary time (%wear time/day) | | |  | |  |
| GT3X+-Norm-150 | 68.4 ± 7.9 | | 65.5 ± 12.7 | | 72.1 ± 10.1 |
| GT3X+-LFE-100 | 60.3 ± 8.7 | | 57.0 ± 14.0 | | 64.8 ± 11.6 |
| GT3X+-LFE-150 | 64.8 ± 8.2 | | 61.7 ± 13.4 | | 68.8 ± 10.9 |
| Breaks (times/day) |  | |  | |  |
| GT3X+-Norm-150 | 80.2 ± 16.6 | | 90.0 ± 25.0 | | 69.2 ± 18.7 |
| GT3X+-LFE-100 | 83.8 ± 16.1 | | 91.8 ± 22.5 | | 75.2 ± 20.4 |
| GT3X+-LFE-150 | 80.8 ± 16 | | 90.1 ± 23.6 | | 70.5 ± 19.6 |
| No. of sedentary bouts ≥2 min (times/day) | |  | |  |  |
| GT3X+-Norm-150 | 54.7 ± 10.7 | | 60.8 ± 14.7 | | 48.4 ± 12.9 |
| GT3X+-LFE-100 | 55.4 ± 10.2 | | 60.0 ± 13.4 | | 51.0 ± 14.3 |
| GT3X+-LFE-150 | 54.3 ± 10.8 | | 60.1 ± 14.1 | | 48.5 ± 14.1 |
| No. of sedentary bouts ≥5 min (times/day) | |  | |  |  |
| GT3X+-Norm-150 | 29.3 ± 6.2 | | 31.6 ± 8.3 | | 27.2 ± 7.6 |
| GT3X+-LFE-100 | 27.9 ± 5.7 | | 28.7 ± 7.8 | | 27.2 ± 8.2 |
| GT3X+-LFE-150 | 28.4 ± 5.8 | | 30.1 ± 7.8 | | 26.9 ± 8.0 |
| No. of sedentary bouts ≥10 min (times/day) | |  | |  |  |
| GT3X+-Norm-150 | 15.8 ± 3.1 | | 16.4 ± 5.4 | | 15.5 ± 4.4 |
| GT3X+-LFE-100 | 13.9 ± 3.5 | | 13.6 ± 5.7 | | 14.4 ± 4.5 |
| GT3X+-LFE-150 | 15.0 ± 3.1 | | 15.2 ± 5.6 | | 14.8 ± 4.3 |
| No. of sedentary bouts ≥ 20 min (times/day) | |  | |  |  |
| GT3X+-Norm-150 | 6.7 ± 2.1 | | 5.9 ± 3.3 | | 7.7 ± 2.9 |
| GT3X+-LFE-100 | 5.4 ± 2.2 | | 4.7 ± 3.0 | | 6.3 ± 3.3 |
| GT3X+-LFE-150 | 6.2 ± 2.1 | | 5.4 ± 3.2 | | 7.1 ± 3.2 |
| No. of sedentary bouts ≥ 30 min (times/day) | |  | |  |  |
| GT3X+-Norm-150 | 3.7 ± 1.6 | | 3.1 ± 2.1 | | 4.4 ± 2.5 |
| GT3X+-LFE-100 | 2.7 ± 1.5 | | 2.2 ± 1.8 | | 3.3 ± 2.6 |
| GT3X+-LFE-150 | 3.4 ± 1.6 | | 2.8 ± 2.1 | | 4.1 ± 2.5 |
| No. of sedentary bouts ≥ 60 min (times/day) | |  | |  |  |
| GT3X+-Norm-150 | 1.0 ± 0.9 | | 0.9 ± 1.1 | | 1.2 ± 1.1 |
| GT3X+-LFE-100 | 0.6 ± 0.7 | | 0.4 ± 0.8 | | 0.8 ± 0.9 |
| GT3X+-LFE-150 | 0.9 ± 0.9 | | 0.6 ± 0.9 | | 1.2 ± 1.2 |
| Data are presented as mean ± SD | | | | | |
| GT3X+, ActiGragh^TM^GT3X+; LFE, low frequency extension | | | | | |
| ^a^ A participant did not answer. | | | | | |
