# Supplementary material for: Comparability of activity monitors used in Asian and Western-country studies for assessing free-living sedentary behaviour
Source: PLoS One. 2017 Oct 18;12(10):e0186523. doi: 10.1371/journal.pone.0186523 (PMC5646850; doi:10.1371/journal.pone.0186523)
Supplement: S2 Table — Values [monitors (A) minus monitors (B)] are mean differences (95% CI). *p < 0.05, **p < 0.01, ***p < 0.001. (DOCX) [file pone.0186523.s004.docx]

**Table S2. Mean differences in sedentary outputs between monitors.** Values [monitors (A) minus monitors (B)] are mean differences (95% CI). *p < 0.05, **p < 0.01, ***p < 0.001

| Total sedentary time (min/day) | | | | |
| --- | --- | --- | --- | --- |
|  |  | Mean Difference (95% CI) | | |
| Monitors (A) | Monitors (B) | Total | Work day | Non-work day |
| GT3X+-Norm-150 | ASP | 122.1 (104.8 to 139.4) *** | 144.6 (123.8 to 165.4) *** | 97.2 (76.1 to 118.3) *** |
|  | AP | 96.5 (75.9 to 117.1) *** | 114.9 (91.4 to 138.4) *** | 83.2 (56.2 to 110.1) *** |
|  | GT3X+-Norm-100 | 32.8 (27.4 to 38.2) *** | 34.8 (25.5 to 44.0) *** | 30.8 (27.2 to 34.5) *** |
|  | GT3X+-LFE-100 | 68.5 (61.7 to 75.2) *** | 75.9 (67.2 to 84.7) *** | 59.0 (51.4 to 66.7) *** |
|  | GT3X+-LFE-150 | 30.7 (26.8 to 34.7) *** | 34.1 (29.4 to 38.9) *** | 26.5 (22.1 to 31) *** |
|  |  |  |  |  |
| GT3X+-LFE-100 | ASP | 53.6 (38.8 to 68.4) *** | 68.6 (51.9 to 85.3) *** | 38.2 (20.6 to 55.7) *** |
|  | AP | 28.1 (7.4 to 48.7) ** | 38.9 (15.4 to 62.5) ** | 24.1 (-0.8 to 49.0) |
|  | GT3X+-Norm-100 | -35.6 (-41.1 to -30.2) *** | -41.2 (-50.1 to -32.2) *** | -28.2 (-32.6 to -23.8) *** |
|  | GT3X+-LFE-150 | -37.7 (-41.0 to -34.5) *** | -41.8 (-46.4 to -37.3) *** | -32.5 (-36.4 to -28.7) *** |
|  |  |  |  |  |
| GT3X+-LFE-150 | ASP | 91.3 (75.7 to 107) *** | 110.4 (92.2 to 128.7) *** | 70.7 (51.9 to 89.4) *** |
|  | AP | 65.8 (45.3 to 86.3) *** | 80.8 (57.7 to 103.8) *** | 56.6 (30.7 to 82.5) *** |
|  | GT3X+-Norm-100 | 2.1 (-2.2 to 6.4) | 0.6 (-7.5 to 8.8) | 4.3 (2.4 to 6.2) *** |

| Total sedentary time (%wear time/day) | | | | |
| --- | --- | --- | --- | --- |
|  |  | Mean Difference (95% CI) | | |
| Monitors (A) | Monitors (B) | Total | Work day | Non-work day |
| GT3X+-Norm-150 | ASP | 14.5 (12.4 to 16.6) *** | 16.2 (14 to 18.5) *** | 12.1 (9.5 to 14.7) *** |
|  | AP | 11.3 (8.9 to 13.7) *** | 13.0 (10.3 to 15.7) *** | 10.0 (6.8 to 13.2) *** |
|  | GT3X+-Norm-100 | 3.9 (3.2 to 4.5) *** | 3.9 (2.8 to 5.0) *** | 3.8 (3.4 to 4.2) *** |
|  | GT3X+-LFE-100 | 8.1 (7.3 to 8.8) *** | 8.5 (7.6 to 9.4) *** | 7.3 (6.5 to 8.2) *** |
|  | GT3X+-LFE-150 | 3.6 (3.2 to 4.1) *** | 3.8 (3.3 to 4.3) *** | 3.3 (2.8 to 3.8) *** |
|  |  |  |  |  |
| GT3X+-LFE-100 | ASP | 6.4 (4.6 to 8.2) *** | 7.7 (5.9 to 9.6) *** | 4.8 (2.5 to 7.0) *** |
|  | AP | 3.2 (0.7 to 5.7) * | 4.5 (1.8 to 7.1) ** | 2.7 (-0.4 to 5.7) |
|  | GT3X+-Norm-100 | -4.2 (-4.9 to -3.6) *** | -4.6 (-5.7 to -3.6) *** | -3.5 (-4.0 to -3.0) *** |
|  | GT3X+-LFE-150 | -4.5 (-4.8 to -4.1) *** | -4.7 (-5.2 to -4.2) *** | -4.0 (-4.5 to -3.6) *** |
|  |  |  |  |  |
| GT3X+-LFE-150 | ASP | 10.9 (8.9 to 12.8) *** | 12.4 (10.4 to 14.4) *** | 8.8 (6.4 to 11.2) *** |
|  | AP | 7.7 (5.2 to 10.1) *** | 9.2 (6.5 to 11.8) *** | 6.7 (3.6 to 9.9) *** |
|  | GT3X+-Norm-100 | 0.2 (-0.3 to 0.8) | 0.1 (-0.9 to 1.0) | 0.5 (0.3 to 0.8) *** |

| Breaks (times/day) | | | | |
| --- | --- | --- | --- | --- |
|  |  | Mean Difference (95% CI) | | |
| Monitors (A) | Monitors (B) | Total | Work day | Non-work day |
| GT3X+-Norm-150 | ASP | 10.4 (5.9 to 15.0) *** | 11.7 (4.2 to 19.1) ** | 8.8 (4.3 to 13.2) *** |
|  | AP | 25.0 (20.4 to 29.5) *** | 25.7 (20.1 to 31.4) *** | 24.6 (19.5 to 29.7) *** |
|  | GT3X+-Norm-100 | -2.8 (-4.5 to -1.2) ** | -1.9 (-4.2 to 0.3) | -4.2 (-5.9 to -2.4) *** |
|  | GT3X+-LFE-100 | -3.6 (-5.9 to -1.3) ** | -1.8 (-5.4 to 1.8) | -6.0 (-8.5 to -3.6) *** |
|  | GT3X+-LFE-150 | -0.6 (-1.9 to 0.8) | -0.1 (-2.2 to 1.9) | -1.3 (-2.7 to 0.0) |
|  |  |  |  |  |
| GT3X+-LFE-100 | ASP | 14.0 (10.0 to 18.0) *** | 13.5 (7.8 to 19.2) *** | 14.8 (10.4 to 19.1) *** |
|  | AP | 28.5 (23.5 to 33.6) *** | 27.6 (21.8 to 33.4) *** | 30.6 (24.3 to 37.0) *** |
|  | GT3X+-Norm-100 | 0.7 (-0.6 to 2.0) | -0.1 (-2.2 to 2.0) | 1.9 (0.6 to 3.2) ** |
|  | GT3X+-LFE-150 | 3.0 (1.7 to 4.3) *** | 1.7 (-0.4 to 3.8) | 4.7 (3.0 to 6.3) *** |
|  |  |  |  |  |
| GT3X+-LFE-150 | ASP | 11.0 (6.8 to 15.2) *** | 11.8 (5.3 to 18.3) *** | 10.1 (5.8 to 14.4) *** |
|  | AP | 25.5 (20.7 to 30.4) *** | 25.9 (20.2 to 31.6) *** | 25.9 (20.2 to 31.7) *** |
|  | GT3X+-Norm-100 | -2.3 (-3.1 to -1.4) *** | -1.8 (-2.8 to -0.8) *** | -2.8 (-3.9 to -1.8) *** |

| No. of sedentary bouts ≥2 min (times/day) | | | | |
| --- | --- | --- | --- | --- |
|  |  | Mean Difference (95% CI) | | |
| Monitors (A) | Monitors (B) | Total | Work day | Non-work day |
| GT3X+-Norm-150 | ASP | 10.0 (7.0 to 13.0) *** | 11.0 (6.3 to 15.7) *** | 8.7 (5.7 to 11.6) *** |
|  | AP | 24.8 (22.1 to 27.6) *** | 26.2 (23.1 to 29.4) *** | 23.8 (20.3 to 27.3) *** |
|  | GT3X+-Norm-100 | -1.1 (-2 to -0.2) * | -0.4 (-1.8 to 1) | -2.1 (-3.2 to -1.1) *** |
|  | GT3X+-LFE-100 | -0.7 (-2.2 to 0.8) | 0.7 (-1.9 to 3.4) | -2.5 (-4.2 to -0.9) ** |
|  | GT3X+-LFE-150 | 0.4 (-0.4 to 1.2) | 0.7 (-0.7 to 2.1) | -0.1 (-1.1 to 0.8) |
|  |  |  |  |  |
| GT3X+-LFE-100 | ASP | 10.7 (8.1 to 13.3) *** | 10.3 (7.0 to 13.6) *** | 11.2 (8.0 to 14.4) *** |
|  | AP | 25.5 (22.7 to 28.4) *** | 25.5 (22.5 to 28.5) *** | 26.3 (22.2 to 30.4) *** |
|  | GT3X+-Norm-100 | -0.4 (-1.3 to 0.5) | -1.1 (-2.8 to 0.6) | 0.4 (-0.8 to 1.6) |
|  | GT3X+-LFE-150 | 1.1 (0.1 to 2.1) * | -0.1 (-1.9 to 1.7) | 2.4 (1.1 to 3.7) *** |
|  |  |  |  |  |
| GT3X+-LFE-150 | ASP | 9.6 (6.7 to 12.4) *** | 10.3 (6.2 to 14.5) *** | 8.8 (5.7 to 11.8) *** |
|  | AP | 24.4 (21.5 to 27.3) ** | 25.5 (22.4 to 28.7) *** | 23.9 (20 to 27.8) *** |
|  | GT3X+-Norm-100 | -1.5 (-2.1 to -1.0) *** | -1.0 (-1.6 to -0.4) *** | -2.0 (-2.8 to -1.2) *** |

| No. of sedentary bouts ≥5 min (times/day) | | | | |
| --- | --- | --- | --- | --- |
|  |  | Mean Difference (95% CI) | | |
| Monitors (A) | Monitors (B) | Total | Work day | Non-work day |
| GT3X+-Norm-150 | ASP | 7.0 (5.5 to 8.5) *** | 8.3 (6.4 to 10.2) *** | 5.8 (4.3 to 7.3) *** |
|  | AP | 9.9 (8.4 to 11.5) *** | 10.4 (8.5 to 12.2) *** | 10.0 (7.9 to 12.1) *** |
|  | GT3X+-Norm-100 | 0.4 (-0.2 to 1.1) | 1.2 (0.2 to 2.1) * | -0.3 (-1 to 0.3) |
|  | GT3X+-LFE-100 | 1.3 (0.4 to 2.3) ** | 2.9 (1.3 to 4.4) *** | 0.0 (-1.1 to 1.1) |
|  | GT3X+-LFE-150 | 0.8 (0.2 to 1.4) ** | 1.4 (0.5 to 2.3) ** | 0.3 (-0.4 to 1.0) |
|  |  |  |  |  |
| GT3X+-LFE-100 | ASP | 5.7 (4.5 to 6.8) *** | 5.4 (4.3 to 6.6) *** | 5.8 (4.1 to 7.4) *** |
|  | AP | 8.6 (7.1 to 10.1) *** | 7.5 (5.9 to 9.1) *** | 10.0 (7.7 to 12.3) *** |
|  | GT3X+-Norm-100 | -0.9 (-1.5 to -0.4) ** | -1.7 (-2.5 to -0.8) *** | -0.3 (-1.1 to 0.5) |
|  | GT3X+-LFE-150 | -0.5 (-1.2 to 0.1) | -1.4 (-2.4 to -0.5) ** | 0.3 (-0.6 to 1.2) |
|  |  |  |  |  |
| GT3X+-LFE-150 | ASP | 6.2 (4.9 to 7.4) *** | 6.9 (5.4 to 8.4) *** | 5.5 (4.0 to 7.0) *** |
|  | AP | 9.1 (7.5 to 10.7) *** | 9.0 (7.4 to 10.5) *** | 9.7 (7.4 to 12.0) *** |
|  | GT3X+-Norm-100 | -0.4 (-0.8 to -0.0) * | -0.2 (-0.7 to 0.3) | -0.6 (-1.1 to -0.1) * |

| No. of sedentary bouts ≥10 min (times/day) | | | | |
| --- | --- | --- | --- | --- |
|  |  | Mean Difference (95% CI) | | |
| Monitors (A) | Monitors (B) | Total | Work day | Non-work day |
| GT3X+-Norm-150 | ASP | 4.4 (3.5 to 5.3) *** | 5.2 (4.1 to 6.3) *** | 3.6 (2.4 to 4.7) *** |
|  | AP | 3.3 (2.4 to 4.2) *** | 3.4 (2.2 to 4.5) *** | 3.6 (2.3 to 4.8) *** |
|  | GT3X+-Norm-100 | 0.9 (0.5 to 1.3) *** | 1.4 (0.8 to 2.1) *** | 0.4 (0.0 to 0.9) |
|  | GT3X+-LFE-100 | 2.0 (1.4 to 2.6) *** | 2.8 (2.0 to 3.6) *** | 1.0 (0.3 to 1.8) * |
|  | GT3X+-LFE-150 | 0.9 (0.6 to 1.2) *** | 1.2 (0.7 to 1.7) *** | 0.6 (0.2 to 1.0) ** |
|  |  |  |  |  |
| GT3X+-LFE-100 | ASP | 2.4 (1.6 to 3.2) *** | 2.4 (1.5 to 3.2) *** | 2.5 (1.3 to 3.7) *** |
|  | AP | 1.4 (0.4 to 2.3) ** | 0.6 (-0.6 to 1.7) | 2.5 (1.4 to 3.7) *** |
|  | GT3X+-Norm-100 | -1.1 (-1.4 to -0.7) *** | -1.4 (-1.9 to -0.9) *** | -0.6 (-1.2 to -0.1) * |
|  | GT3X+-LFE-150 | 1.1 (0.6 to 1.5) *** | 1.6 (1.1 to 2.2) *** | 0.4 (-0.2 to 1.0) |
|  |  |  |  |  |
| GT3X+-LFE-150 | ASP | 3.5 (2.7 to 4.3) *** | 4.0 (3.0 to 5.0) *** | 2.9 (1.8 to 4.0) *** |
|  | AP | 2.5 (1.6 to 3.3) *** | 2.2 (1.1 to 3.3) *** | 3.0 (1.8 to 4.1) *** |
|  | GT3X+-Norm-100 | 0.0 (-0.2 to 0.3) | 0.2 (-0.1 to 0.6) | -0.2 (-0.5 to 0.1) |

| No. of sedentary bouts ≥20 min (times/day) | | | | |
| --- | --- | --- | --- | --- |
|  |  | Mean Difference (95% CI) | | |
| Monitors (A) | Monitors (B) | Total | Work day | Non-work day |
| GT3X+-Norm-150 | ASP | 1.5 (0.9 to 2.0) *** | 1.5 (0.9 to 2.1) *** | 1.4 (0.6 to 2.1) *** |
|  | AP | 0.5 (0 to 1.0) * | 0.2 (-0.4 to 0.8) | 0.8 (0.2 to 1.5) * |
|  | GT3X+-Norm-100 | 0.8 (0.6 to 1.1) *** | 0.7 (0.4 to 1) *** | 0.9 (0.5 to 1.3) *** |
|  | GT3X+-LFE-100 | 1.3 (1 to 1.7) *** | 1.2 (0.9 to 1.5) *** | 1.5 (0.9 to 2.0) *** |
|  | GT3X+-LFE-150 | 0.6 (0.3 to 0.8) *** | 0.5 (0.3 to 0.8) *** | 0.6 (0.3 to 0.9) *** |
|  |  |  |  |  |
| GT3X+-LFE-100 | ASP | 0.1 (-0.4 to 0.7) | 0.3 (-0.2 to 0.9) | -0.1 (-0.9 to 0.7) |
|  | AP | -0.8 (-1.4 to -0.3) ** | -1.0 (-1.6 to -0.3) ** | -0.6 (-1.4 to 0.1) |
|  | GT3X+-Norm-100 | -0.5 (-0.7 to -0.3) *** | -0.5 (-0.7 to -0.2) *** | -0.6 (-0.9 to -0.3) *** |
|  | GT3X+-LFE-150 | -0.8 (-1.1 to -0.5) *** | -0.7 (-1.0 to -0.3) *** | -0.9 (-1.3 to -0.5) *** |
|  |  |  |  |  |
| GT3X+-LFE-150 | ASP | 0.9 (0.3 to 1.5) ** | 1.0 (0.3 to 1.6) ** | 0.8 (0.0 to 1.5) * |
|  | AP | -0.1 (-0.6 to 0.4) | -0.3 (-0.9 to 0.3) | 0.3 (-0.4 to 0.9) |
|  | GT3X+-Norm-100 | 0.3 (0.1 to 0.4) ** | 0.2 (0.0 to 0.3) * | 0.3 (0.0 to 0.6) * |

| No. of sedentary bouts ≥30 min (times/day) | | | | |
| --- | --- | --- | --- | --- |
|  |  | Mean Difference (95% CI) | | |
| Monitors (A) | Monitors (B) | Total | Work day | Non-work day |
| GT3X+-Norm-150 | ASP | 0.9 (0.4 to 1.3) *** | 1.0 (0.6 to 1.5) *** | 0.7 (0.1 to 1.2) * |
|  | AP | -0.1 (-0.5 to 0.4) | -0.1 (-0.7 to 0.4) | 0.0 (-0.5 to 0.5) |
|  | GT3X+-Norm-100 | 0.5 (0.3 to 0.7) *** | 0.5 (0.3 to 0.7) *** | 0.5 (0.2 to 0.7) *** |
|  | GT3X+-LFE-100 | 1.0 (0.8 to 1.3) *** | 1.0 (0.7 to 1.3) *** | 1.0 (0.7 to 1.3) *** |
|  | GT3X+-LFE-150 | 0.3 (0.2 to 0.5) *** | 0.4 (0.2 to 0.6) *** | 0.2 (0.1 to 0.4) ** |
|  |  |  |  |  |
| GT3X+-LFE-100 | ASP | -0.2 (-0.6 to 0.2) | 0.1 (-0.3 to 0.5) | -0.4 (-0.9 to 0.2) |
|  | AP | -1.1 (-1.5 to -0.6) *** | -1.1 (-1.6 to -0.6) *** | -1.0 (-1.5 to -0.5) *** |
|  | GT3X+-Norm-100 | -0.5 (-0.7 to -0.4) *** | -0.4 (-0.6 to -0.2) *** | -0.6 (-0.8 to -0.4) *** |
|  | GT3X+-LFE-150 | -0.7 (-0.9 to -0.5) *** | -0.6 (-0.8 to -0.3) *** | -0.8 (-1.0 to -0.6) *** |
|  |  |  |  |  |
| GT3X+-LFE-150 | ASP | 0.5 (0.1 to 1.0) ** | 0.6 (0.2 to 1.1) ** | 0.4 (-0.2 to 1.0) |
|  | AP | -0.4 (-0.8 to 0.0) | -0.5 (-1.0 to 0.0) * | -0.3 (-0.8 to 0.3) |
|  | GT3X+-Norm-100 | 0.2 (0.1 to 0.3) ** | 0.1 (0.0 to 0.3) | 0.2 (0.1 to 0.4) * |

| No. of sedentary bouts ≥60 min (times/day) | | | | |
| --- | --- | --- | --- | --- |
|  |  | Mean Difference (95% CI) | | |
| Monitors (A) | Monitors (B) | Total | Work day | Non-work day |
| GT3X+-Norm-150 | ASP | 0.2 (0.0 to 0.5) | 0.4 (0.2 to 0.6) *** | 0.1 (-0.3 to 0.4) |
|  | AP | -0.2 (-0.5 to 0.0) * | 0.1 (-0.1 to 0.3) | -0.6 (-0.9 to -0.3) *** |
|  | GT3X+-Norm-100 | 0.2 (0.1 to 0.3) *** | 0.3 (0.1 to 0.4) *** | 0.2 (0.1 to 0.3) ** |
|  | GT3X+-LFE-100 | 0.5 (0.3 to 0.6) *** | 0.4 (0.2 to 0.7) *** | 0.5 (0.3 to 0.7) *** |
|  | GT3X+-LFE-150 | 0.1 (0.1 to 0.2) ** | 0.2 (0.1 to 0.3) ** | 0.1 (0.0 to 0.2) |
|  |  |  |  |  |
| GT3X+-LFE-100 | ASP | -0.2 (-0.5 to 0.0) | 0.0 (-0.3 to 0.2) | -0.4 (-0.8 to 0.0) |
|  | AP | -0.7 (-0.9 to -0.5) *** | -0.3 (-0.5 to -0.1) ** | -1.1 (-1.4 to -0.7) *** |
|  | GT3X+-Norm-100 | -0.2 (-0.3 to -0.1) *** | -0.2 (-0.3 to 0.0) * | -0.3 (-0.4 to -0.1) *** |
|  | GT3X+-LFE-150 | -0.3 (-0.4 to -0.2) *** | -0.2 (-0.4 to -0.1) ** | -0.4 (-0.6 to -0.2) *** |
|  |  |  |  |  |
| GT3X+-LFE-150 | ASP | 0.1 (-0.2 to 0.3) | 0.2 (0.0 to 0.4) | 0.0 (-0.4 to 0.4) |
|  | AP | -0.4 (-0.6 to -0.1) ** | -0.1 (-0.3 to 0.1) | -0.7 (-1 to -0.3) *** |
|  | GT3X+-Norm-100 | 0.1 (0.0 to 0.1) * | 0.0 (0.0 to 0.1) | 0.1 (0.0 to 0.3) * |
